# 3D deuterium metabolic imaging (DMI) of the human liver at 7 T using low‐rank and subspace model‐based reconstruction

**DOI:** 10.1002/mrm.30395

**Published:** 2024-12-22

**Authors:** Kyung Min Nam, Ayhan Gursan, Nam G. Lee, Dennis W. J. Klomp, Jannie P. Wijnen, Jeanine J. Prompers, Arjan D. Hendriks, Alex A. Bhogal

**Affiliations:** ^1^ Center for Image Sciences, High Field MR Research Group, Department of Radiology University Medical Centre Utrecht Utrecht The Netherlands; ^2^ Alfred E. Mann Department of Biomedical Engineering University of Southern California Los Angeles California USA

**Keywords:** ^2^H, 7 T, deuterium, deuterium MRSI, DMI, liver, low rank, SPICE, subspace model

## Abstract

**Purpose:**

To implement a low‐rank and subspace model‐based reconstruction for 3D deuterium metabolic imaging (DMI) and compare its performance against Fourier transform‐based (FFT) reconstruction in terms of spectral fitting reliability.

**Methods:**

Both reconstruction methods were applied on simulated and experimental DMI data. Numerical simulations were performed to evaluate the effect of increasing acceleration factors. The impact on spectral fitting results, SNR, and the overall normalized root mean square error (NRMSE) compared to ground‐truth data were calculated. A comparative analysis was performed on DMI data acquired from the human liver, including both natural abundance and post‐deuterated glucose intake data at 7 T.

**Results:**

Simulation showed the Cramer‐Rao lower bound [%] of water, glucose, sum of glutamate and glutamine (Glx), and lipid signals for the low‐rank and subspace model‐based reconstruction at R = 1.0 was 12.4, 14.7, 17.3, and 11.0 times lower than FFT. At R = 1.1, NRMSE was 1.4%, 1.3%, 0.8%, and 4.2% lower for the water, glucose, Glx, and lipid, respectively, compared to FFT. However, the NRMSE of the Glx and lipid increased by 0.4% and 3.2% at R = 1.3. For the in vivo DMI experiment, SNR was 2.5–3.0 times higher compared to FFT. The fitted amplitude of water and glucose peaks showed Cramer‐Rao lower bound [%] values that were approximately 2.3 times lower than FFT.

**Conclusion:**

Simulations and in vivo experiments on the human liver demonstrate that low‐rank and subspace model‐based reconstruction with undersampled data mitigates noise and enhances spectral fitting quality.

## 1 INTRODUCTION

Deuterium (i.e., D or ^2^H) metabolic imaging^1^, ^2^ (DMI) is an emerging technique for noninvasive metabolic imaging. DMI is used to spatially map metabolism in vivo by integrating deuterium magnetic resonance spectroscopic imaging (MRSI) with the intake of deuterium‐labeled substrates such as [6,6'‐^2^H_2_]‐glucose. The deuterium‐labeled substrate can be introduced into laboratory animals or the human body, allowing for monitoring of the substrate and its downstream labeled metabolites (i.e., glutamate and glutamine [Glx], and lactate) using deuterium MRSI. DMI experiments^1^, ^3^, ^4^ have been effective in evaluating metabolic rates and metabolic shifts between glycolysis and oxidation in brain tumors (e.g., the Warburg effect^5^, ^6^).

Conventional ^1^H MRSI methods encounter significant limitations, including low spatial resolution, low signal‐to‐noise ratio (SNR), and long acquisition times. These challenges become more pronounced when applied to X‐nuclei due to its lower intrinsic sensitivity than ^1^H. To overcome these difficulties, several efforts^7^, ^8^ have focused on developing rapid sequences and advanced reconstruction techniques^9^, ^10^, ^11^, ^12^, ^13^, ^14^, ^15^, ^16^, ^17^, ^18^, ^19^, ^20^, ^21^, ^22^, ^23^, ^24^, ^25^, ^26^, ^27^, ^28^, ^29^, ^30^, ^31^, ^32^, ^33^, ^34^, ^35^, ^36^, ^37^ that can provide high‐resolution data with sufficient SNR for spectral analysis.

One promising approach is SPICE^38^, ^39^, ^40^, ^41^, ^42^, ^43^, ^44^ (spectroscopic imaging by exploiting spatiospectral correlation), which acquires two complementary datasets for different purposes: (1) a dataset sampled from the center of k‐space with limited spatial coverage but fully sampled coverage in the temporal dimension, and (2) a dataset acquired with a variant of the echo‐planar readout that has a large spatial coverage but limited coverage in the temporal dimension. SPICE includes reconstruction strategies based on low‐rank^45^ and subspace modeling^46^, ^47^, ^48^ to attain high SNR during rapid acquisition while recovering high‐dimensional spectroscopic data.

The SPICE framework, which exploits the spatiotemporal partial separability^45^ of high‐dimensional spectroscopic signals, has been demonstrated in various applications for different nuclei,^38^, ^39^, ^40^, ^41^, ^49^, ^50^, ^51^, ^52^, ^53^, ^54^, ^55^ including rodent hind limbs^54^ and human brain.^49^, ^55^ However, to the best of our knowledge, SPICE has not been applied to DMI of the human liver. In body DMI, acquiring a dynamic off‐resonance map during free‐breathing is highly challenging. Nevertheless, given the relatively short T_2_* in the liver, we assume that line broadening is dominant and the effects of static off‐resonances are minimal. This allows us to implement SPICE without the need for off‐resonance correction, making it an attractive approach for DMI. In this study, we aim to investigate the feasibility of a low‐rank and subspace model‐based reconstruction, a core component of the SPICE framework, combined with total variation regularization, for DMI of the human liver.

## 2 THEORY

### 2.1 Signal model

In MRSI, the measured k‐space data for the cth coil (*c* = 1, …, $N_{c}$) over the region of interest (ROI) V and spectral bandwidth $\Omega_{f}$ can be expressed as:

$$d_{c}\left(k,t\right)=\int_{V}\int_{\Omega_{f}}s_{c}\left(r\right)\;\rho \left(r,f\right)\;e^{-j2\pi \text{ft}}e^{-j2\pi k\cdot r}\;\mathrm{dfd}r+\xi_{c}\left(k,t\right)$$

(1) $$=\int_{V}s_{c}\left(r\right)\;\tilde{\rho }\left(r,t\right)\;e^{-j2\pi k\cdot r}\;dr+\xi_{c}\left(k,t\right),$$

where $N_{c}$ is the total number of coils, ρ(r,f) denotes the desired spatiospectral function representing the spatial r and spectral information f of the object. $s_{c}\left(r\right)$ is the receive coil sensitivity at position r of the cth coil, and k represents the coordinates for the Fourier encoding space. $\tilde{\rho }\left(r,t\right)$ denotes the spatiotemporal function, and the measurement noise $\xi_{c}$ is assumed to be complex white Gaussian.

### 2.2 Subspace modeling

The low‐rank and subspace model exploits spatiotemporal correlation of the MRSI signals:

(2) $$\tilde{\rho }\left(r,t\right)=\sum_{\ell =1}^{L}u_{\ell }\left(r\right)v_{\ell }\left(t\right),$$

where L is the model order, and ${\left\{u_{\ell }\left(r\right)\right\}}_{\ell =1}^{L}$ and ${\left\{v_{\ell }\left(t\right)\right\}}_{\ell =1}^{L}$ represent spatial coefficients and temporal basis functions, respectively. The Casorati matrix $C\in {\mathbb{C}}^{N\times M}$ of the spatiotemporal function $\tilde{\rho }\left(r,t\right)$ is defined as.

(3) $$\begin{array}{cc} C≜ & \left[\begin{array}{ccc} \tilde{\rho }\left(r_{1},t_{1}\right) & \cdots & \tilde{\rho }\left(r_{1},t_{M}\right)\\ \vdots & \ddots & \vdots \\ \tilde{\rho }\left(r_{N},t_{1}\right) & \cdots & \tilde{\rho }\left(r_{N},t_{M}\right) \end{array}\right]\\ = & \underset{U}{\underbrace{\left[\begin{array}{ccc} u_{1}\left(r_{1}\right) & \cdots & u_{L}\left(r_{1}\right)\\ \vdots & \ddots & \vdots \\ u_{1}\left(r_{N}\right) & \cdots & u_{L}\left(r_{N}\right) \end{array}\right]}}\;\underset{V}{\underbrace{\left[\begin{array}{ccc} v_{1}\left(t_{1}\right) & \cdots & v_{1}\left(t_{M}\right)\\ \vdots & \ddots & \vdots \\ v_{L}\left(t_{1}\right) & \cdots & v_{L}\left(t_{M}\right) \end{array}\right]}}, \end{array}$$

where N is the number of voxels, and M is the number of time samples. The columns of $U\in {\mathbb{C}}^{N\times L}$ and rows of $V\in {\mathbb{C}}^{L\times M}$ span the spatial and temporal subspaces of the Casorati matrix, respectively. The spatiotemporal function $\tilde{\rho }\left(r,t\right)$ is inherently low rank (i.e., small L) because each spectrum can be synthetized from a weighted combination of a limited number of metabolites. A discrete signal model for Equation 1 can be written as:

(4) $$d_{c}=\Omega \left(FS_{c}C\right)+\xi_{c},$$

where $d_{c}\in {\mathbb{C}}^{N_{k}\times 1}$ is the vector representation of measured k‐space data, $S_{c}\in {\mathbb{C}}^{N\times N}$ is a diagonal matrix containing the coil sensitivities of the cth coil, $F\in {\mathbb{C}}^{N\times N}$ denotes the Fourier encoding matrix, and $\Omega \left(\cdot \right):{\mathbb{C}}^{N\times M}\to {\mathbb{C}}^{N_{k}\times 1}$ denotes a subsampling operator that selects only measured data.

The low‐rank and subspace model‐based reconstruction framework solves the following minimization problem^47^:

(5) $$\hat{U}=\underset{U}{\text{argmin}}\;\sum_{c=1}^{N_{c}}{\left\|d_{c}-\Omega \left(FS_{c}U\hat{V}\right)\right\|}_{\ell_{2}}^{2}+\lambda \;{\left\|\mathrm{DU}\right\|}_{\ell_{1}},$$

where D = $\left[\begin{array}{l} D_{x}\\ D_{y}\\ D_{z} \end{array}\right]$ is a finite difference operator with $D_{x},D_{y},$ and $D_{z}$ being the derivatives in the x, y, and z directions in the spatial domain, respectively.

We use a primal‐dual hybrid gradient algorithm^56^ to solve a total variation–constrained optimization problem Equation (5) implemented in the *pics* command of Berkeley Advanced Reconstruction Toolbox (BART)^57^ with “‐B file” option^58^ to enforce temporal subspace.

## 3 METHODS

### 3.1 In vivo experimental setup

The 7 T MR (Philips Medical Systems, Best, The Netherlands) experiments were performed using a ^1^H/^2^H transmit–receive body array,^10^, ^59^ consisting of four proton dipole antennas and four deuterium loop coils. A ^1^H B_0_ map was acquired for second‐order image‐based B_0_ shimming.^60^ Anatomical ^1^H T_1_‐weighted images using a Dixon method were obtained to plan the DMI acquisition.

### 3.2 Image reconstruction

The raw data were first converted from the vendor's proprietary format to a.hdr/cfl file (used in BART) and read in MatLab R2021b (MathWorks, Natick, MA). The BART commands were called within MatLab on a laptop PC equipped with one 2.80 GHz four‐core Intel Xeon E3‐1505M processor (Santa Clara, CA) and 32 GB RAM. The reconstruction of one dataset took approximately 30 s with 30 primal‐dual iterations.

The following steps were used to calculate a pre‐estimated temporal subspace $\hat{V}$: (1) The coil sensitivity maps (CSM) (e.g., 12 × 18 × 15) were estimated with ESPIRiT^61^ calibration using the ecalib command of BART; (2) the estimated CSMs were transformed into k‐space; (3) a central region of k‐space was extracted (e.g., 3 × 5 × 3) and (4) transformed back into image space; (5) each voxel in CSMs was normalized according to Equation (2),^61^ that is, $\hat{S}={\left[\sum_{c=1}^{N_{c}}S_{c}^{H}S_{c}\right]}^{-1/2}S$; (6) the raw data in k‐space was processed by extracting a region of size D1, which refers to the fully sampled central k‐space region used for temporal basis estimation, as described in steps 3 and 4, and was followed by transformation into image space; (7) channel combination was performed in image space; (8) a Casorati matrix $C\in {\mathbb{C}}^{N\times M}$ was formed; and (9) the singular value decomposition^38^, ^45^, ^46^, ^47^ was applied to the Casorati matrix and then $\hat{V}\in {\mathbb{C}}^{L\times M}$ was determined with its L principal right singular vectors. Using a pre‐estimated temporal subspace reduces the number of unknowns from N×M to L×M, making the reconstruction well‐conditioned. The normalized root mean square error (NRMSE) of the spectra and the Cramer‐Rao lower bound (CRLB [%]) of the fitted metabolite signals were calculated as a function of the regularization parameter λ, which was set to 0.001 (Figure S1).

### 3.3 Numerical simulations

**FIGURE 1:**
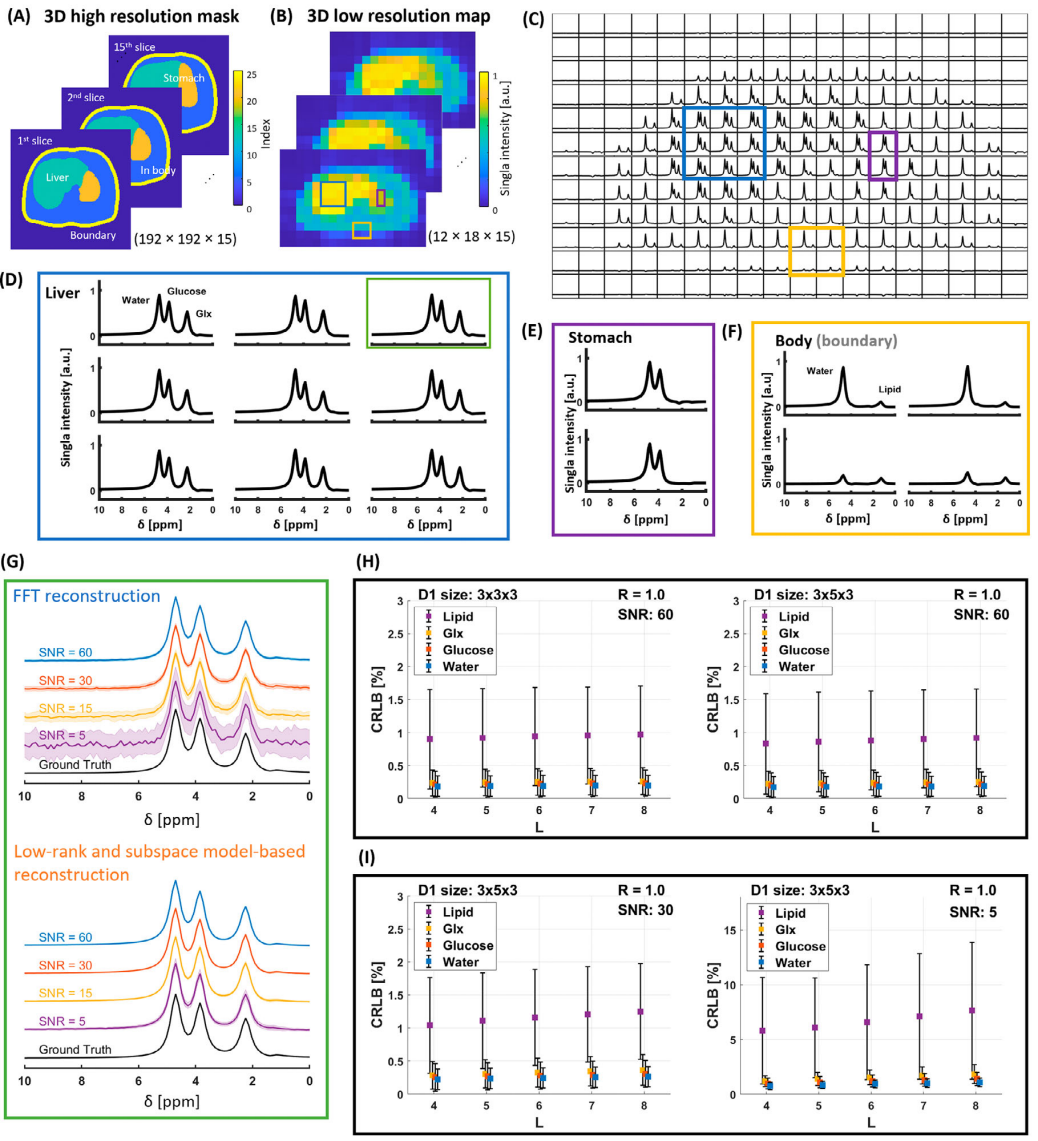
Schematic overview of the numerical simulation setup depicting the ROIs, along with representative spectra obtained from each region. (A) 3D high‐resolution phantom mask was transformed to 3D k‐space and truncated to a lower resolution (B). (C) In the deuterium spectra, ROI are shown, including the liver (blue box), stomach (purple box), and the boundary of the body (yellow box). Metabolite signals for water, glucose, Glx, and lipid (chemical shifts: 4.7 ppm, 3.8 ppm, 2.25 ppm, and 1.3 ppm) were generated. (D, E, F) Representative spectra in each ROI: deuterated water, glucose, and Glx signals are shown in the liver (D); water and glucose in the stomach (E); and water in the body, and water and lipids (i.e., subcutaneous fat) in the boundary of the body (F). (G) The spectra from a voxel in the liver ROI (green box in D) are shown for different SNR levels (60, 30, 15, and 5) using both FFT reconstruction and low‐rank and subspace model‐based reconstruction. The ground truth spectrum is displayed at the bottom for comparison. Noise was added to the ground truth data to simulate the four different SNR conditions. The figure illustrates how increasing noise levels affect spectral quality, with the low‐rank and subspace model‐based reconstruction maintaining better spectral fidelity at lower SNRs compared to FFT reconstruction. (H) To determine the optimal D1 size, the CRLB [%] was calculated as the model order L increased for metabolites at two different D1 sizes (3 × 3 × 3 and 3 × 5 × 3), with an acceleration factor of R = 1.0 and SNR = 60. The CRLB for lipid was the highest among all metabolites, followed by Glx, glucose, and water. The results show that the CRLB values were lower for D1 size 3 × 5 × 3 compared to 3 × 3 × 3. (I) The effect of decreasing SNR on CRLB is shown for D1 size 3 × 5 × 3. As SNR decreases (e.g., SNR = 60, 30, and 5), the CRLB values for all metabolites increase, demonstrating the impact of noise on estimation precision. CRLB, Cramer‐Rao lower bound; FFT, Fourier transform‐based; Glx, sum of glutamate and glutamine; ROI, region of interest.

To elucidate how the D1 sizes and model orders impact the CRLB of fitted metabolite signals in the low‐rank and subspace model‐based reconstruction method, as well as the NRMSE under an undersampling condition, numerical simulations were conducted. The human liver model utilized in this simulation was derived from the 4D extended cardiac‐torso numerical phantom (XCAT^62^, ^63^). A 3D high‐resolution mask of human organs was generated based on the extended cardiac‐torso phantom, containing three distinct compartments: the liver; stomach; and the rest of the body, including a subcutaneous fat layer. An associated high‐resolution image was generated (matrix size: 256 × 256 × 15) to accurately depict these anatomical compartments (Figure 1A).

Metabolite signals for deuterated water, glucose, Glx, and lipid (subcutaneous fat) were generated using a Lorentzian line shape with a linewidth of 30 Hz^64^ and a relative amplitude of 1.0 (water reference), 0.8, 0.6, and 0.6, respectively. Complex white Gaussian noise was independently added to each channel, generating noisy datasets with SNRs of 60, 30, 15, and 5. The SNR was verified by 10,000 repetitions using the set noise standard deviation (SD), with SNR = 15 selected for its similarity to in vivo experiments. The simulation study involved reconstructing and fitting noisy data using two methods (low‐rank and subspace model‐based reconstruction; and Fourier transform‐based [FFT] reconstruction) and calculating the CRLB and NRMSE. To ensure consistent noise distribution at the selected SNR, we conducted 30 repetitions for each dataset using the same noise distribution.

The simulated spectra were assigned a spectral bandwidth of 5 kHz with 1024 time‐points. The 3D high‐resolution images were converted to the k‐space domain using a Fourier transform, and the k‐space data were center‐extracted to fit a 12 × 18 × 15 matrix (Figure 1B). This down‐sampling process aligned the high‐resolution images with the resolution commonly used in DMI acquisitions. Low‐rank and subspace model‐based reconstruction was then applied to determine temporal subspace ${\left\{v_{l}\left(t\right)\right\}}_{l=1}^{L}$ for different D1 sizes from the limited central k‐space (i.e., 3 × 3 × 3 and 3 × 5 × 3). The selection of the model order L (Figure S2) involves determining how many metabolites are included in the simulation data, organizing the data into a Casorati matrix, and applying singular value decomposition to identify the singular values.

**FIGURE 2:**
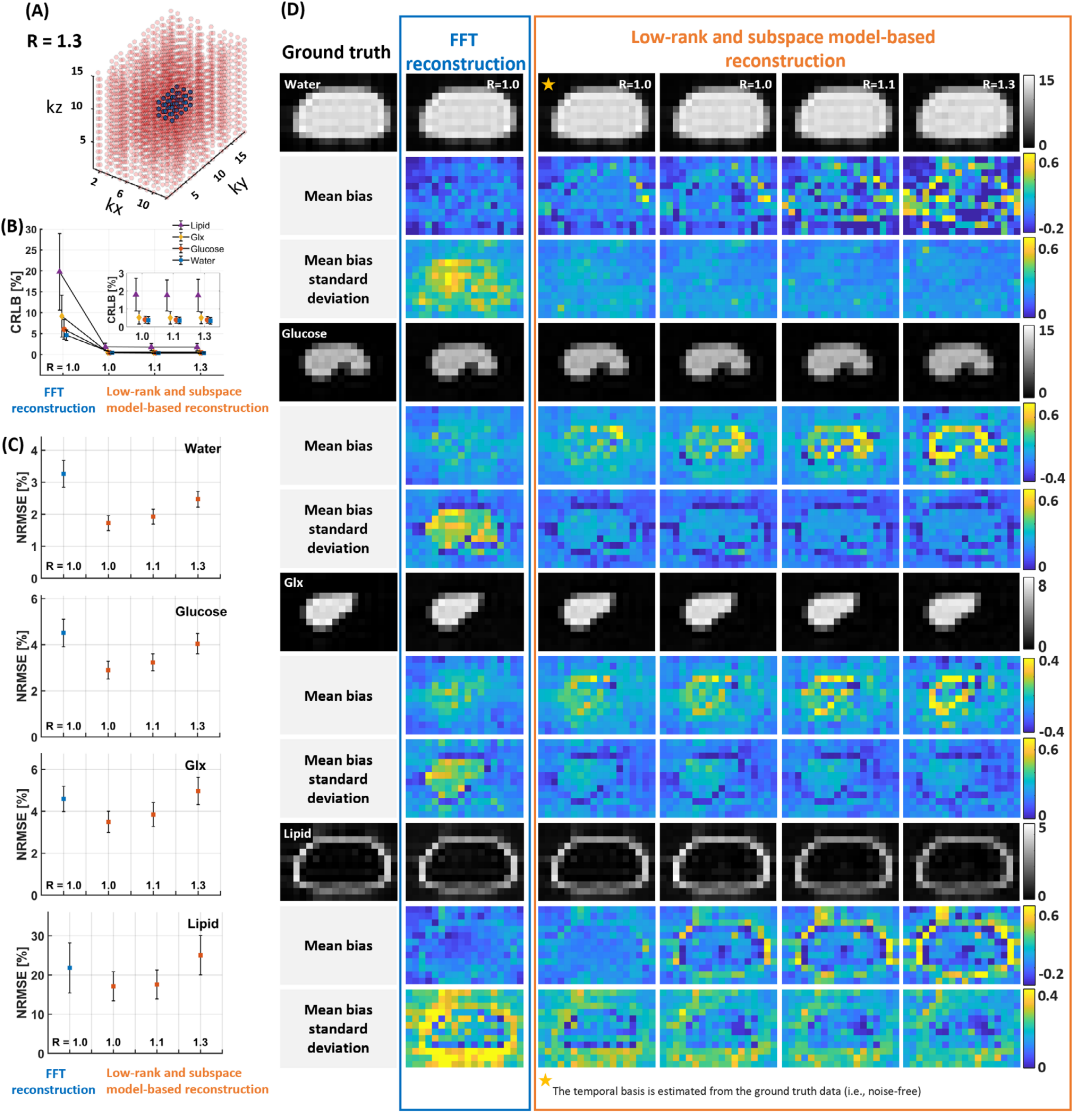
(A) The Poisson disk sampling pattern (red circles) is shown under undersampling conditions at an acceleration factor of R = 1.3. The sampling data (i.e., D1 size = 3 × 5 × 3) of the center (dark blue circles) is used for temporal basis estimation. (B) The changes in CRLB [%] for FFT reconstruction and low‐rank and subspace model‐based reconstruction with acceleration factors of R = 1.0, 1.1, and 1.3 are presented using noisy data with SNR = 15, with a zoomed view for the low‐rank and subspace model‐based reconstruction. (C) The NRMSE of the fitted signals for each metabolite was calculated for both FFT reconstruction and low‐rank and subspace model‐based reconstruction at acceleration factors of R = 1.0, 1.1, and 1.3. (D) Metabolite maps were created from the fitted signals for ground truth, FFT reconstruction, and low‐rank and subspace model‐based reconstruction. The first column shows the ground truth metabolite maps: The first row shows water, the fourth row presents glucose, the seventh row displays Glx, and the tenth row depicts lipid. The second column displays the metabolite maps from FFT reconstruction, along with the corresponding mean bias and mean bias SD maps. The third column presents the results from low‐rank and subspace model‐based reconstruction at R = 1.0, where the temporal basis was estimated from the ground truth. Columns four through six show the metabolite maps, mean bias maps, and mean bias SD maps for low‐rank and subspace model‐based reconstruction at R = 1.0, 1.1, and 1.3, respectively. NRMSE, normalized root mean square error.

Signal fitting was performed with SNR = 15 for both reconstructions, and average metabolite maps were generated for each metabolite (Figure 2D). To assess bias, the mean bias maps were created by subtracting the signal‐fitted metabolite maps from the ground truth and calculating the average across 30 datasets. Mean bias SD maps were also generated by calculating the SD of the differences between the fitted and ground truth maps.

### 3.4 Deuterium MRSI acquisition of in vivo data

This investigation was conducted according to the Declaration of Helsinki (2013 version) and approved by the ethics committee of the University Medical Center Utrecht (institutional review board number: 21–122). Participants provided written informed consent. Deuterium MRSI data was acquired from the livers of two healthy volunteers. The first volunteer was scanned at the natural abundance, whereas the second was scanned 2 h after orally ingesting deuterated glucose 50 g of [6,6'‐^2^H_2_] glucose (≥99% purity) dissolved in water following an overnight fast.

For the acquisition of deuterium MRSI data in the human liver, both at natural abundance and after oral intake of deuterated glucose, a 3D MRSI sequence with Hamming‐weighted acquisition and four averages was utilized. Afterward, a correction was applied to the weighting coefficients for the k‐space locations to align the discrete number of acquired averages with the ideal Hamming function.^10^, ^59^ Datasets retrospectively accelerated from R = 1.0 to 1.3 were used to calculate the SNR and CRLB [%]. The scan parameters for the in vivo experiments are provided in Table S1.

### 3.5 Quantification

The noise was added to the ground truth data based on the following equations. For each noise level n, the noise is added to the spatial‐spectral data as follows:

(6) $$S_{n}^{\prime }=S+\xi_{n}$$

(7) ξn=αn×1√2(Re(N(0,1))+i×Im(N(0,1))),

where $S_{n}^{\prime }$ is the added‐noise data (x,y,z,f), S is the noise‐free data, $\xi_{n}$ is the noise data, $\alpha_{n}$ is a constant for given noise n, i is the imaginary unit, and N(0,1) represents a complex Gaussian noise with real and imaginary parts drawn from a normal distribution with mean 0 and variance 1.

The SNR was computed using the equation SNR = $S_{n,\text{water}}^{\prime }$/ σ($S_{n,\text{noise}}^{\prime }$), where $S_{n,\text{water}}^{\prime }$ represents the water signal intensity and σ($S_{n,\text{noise}}^{\prime }$) is the SD of the noise within the 8–12 ppm range. The noisy datasets were determined to correspond to the mean SNR values of 60.0 ± 7.3, 30.4 ± 3.8, 15.1 ± 2.1, and 5.1 ± 1.2, respectively. The $\alpha_{n}$ values (i.e., $\alpha_{1}=$ 0.923, $\alpha_{2}=$ 1.830, $\alpha_{3}=$ 3.700, and $\alpha_{4}=$ 10.900) were chosen by adding random noise and verifying that the resulting SNR values matched the desired levels after 10,000 repetitions. In both experiments, the calculated SNR was compared in the spectra of the ROI after applying both reconstructions. A noise‐free dataset was utilized as a reference for the simulations.

Metabolite maps were generated using the advanced method for accurate, robust, and efficient spectral fitting (AMARES) algorithm,^65^ implemented within the open‐source magnetic resonance spectroscopy analysis (OXSA) toolbox,^66^ for signal fitting. The CRLB was computed for each metabolite signal to estimate the fitting error of metabolic concentration estimates. To assess the accuracy of generated metabolite maps, we use the overall NRMSE (= ${\left\|\theta -\hat{\theta }\right\|}_{2}/{\left\|\theta \right\|}_{2}$), where θ and $\hat{\theta }$, respectively, denote the metabolite signal amplitude fit from the reference and reconstruction.

## 4 RESULTS

### 4.1 Numerical Simulation

In the simulation, noise‐free deuterium signals were generated in the high‐resolution map and down‐sampled to a 12 × 18 × 15 matrix. Unlike the distinct organ shapes in high‐resolution masks (Figure 1A), the low‐resolution maps (Figure 1B) lacked clear delineation. Examining the ^2^H spectra in the low‐resolution map reveals the presence of metabolite signals defined by each organ such as liver and stomach (Figure 1D,E). The resulting SNR from FFT reconstruction (Figure 1G) was 60, 30, 15, and 5, respectively.

The CRLB was calculated for low‐rank and subspace model‐based reconstruction data with SNR = 60 for different model orders (L = 4 to 8) and D1 sizes (3 × 3 × 3 and 3 × 5 × 3). The results show that the CRLB was lower for a D1 size of 3 × 5 × 3 compared to 3 × 3 × 3 (Figure 1H). Specifically at L = 5, the CRLB for glucose and lipid was 0.1% lower for the 3 × 5 × 3 size. Moreover, the CLRB was computed for a D1 size of 3 × 5 × 3 at different SNRs, ranging from 30 to 5 (Figure 1I). The CRLB for water increased by a factor of 3, for glucose and Glx by a factor of 4, and for lipid by a factor of 5.6.

CRLB and NRMSE were calculated for both reconstructions using retrospectively undersampled data at acceleration factors R = 1.0, 1.1, and 1.3 (Figure 2B,C). CRLB values from FFT reconstruction were higher than those from low‐rank and subspace model‐based reconstruction across all metabolites when SNR = 15. The CRLB was 12.4 times higher for water, 14.7 times for glucose, 17.3 times for Glx, and 11.0 times for lipid. The CRLB values for low‐rank and subspace model‐based reconstruction remained stable across the acceleration factors, R = 1.0, 1.1, and 1.3.

The NRMSE values for each metabolite were as follows: For water, the NRMSE was 3.3 ± 0.4 for FFT and 1.7 ± 0.2 (R = 1.0), 1.9 ± 0.2 (R = 1.1), and 2.5 ± 0.2 (R = 1.3) for low‐rank and subspace model‐based reconstruction. For glucose, the NRMSE was 4.5 ± 0.6 for FFT and 2.9 ± 0.4 (R = 1.0), 3.2 ± 0.4 (R = 1.1), and 4.0 ± 0.4 (R = 1.3). Similarly, for Glx, the NRMSE was 4.6 ± 0.6 for FFT and 3.5 ± 0.5 (R = 1.0), 3.8 ± 0.6 (R = 1.1), and 5.0 ± 0.6 (R = 1.3). For lipid, the NRMSE was 21.8 ± 6.4 for FFT, and 17.1 ± 3.7 (R = 1.0), 17.6 ± 3.7 (R = 1.1), and 25.0 ± 5.0 (R = 1.3) for low‐rank and subspace model‐based reconstruction.

Notably, the metabolite maps generated by extracting the temporal basis from the ground truth in the low‐rank and subspace model‐based reconstruction presented less mean bias than those generated from the noisy datasets. However, in the low‐rank and subspace model‐based reconstruction, the mean bias increased with higher acceleration factors and became slightly higher than in the FFT reconstruction. Despite this, the metabolite maps from low‐rank and subspace model‐based reconstruction still showed lower mean bias SD compared to FFT reconstruction.

### 4.2 In vivo

**FIGURE 3:**
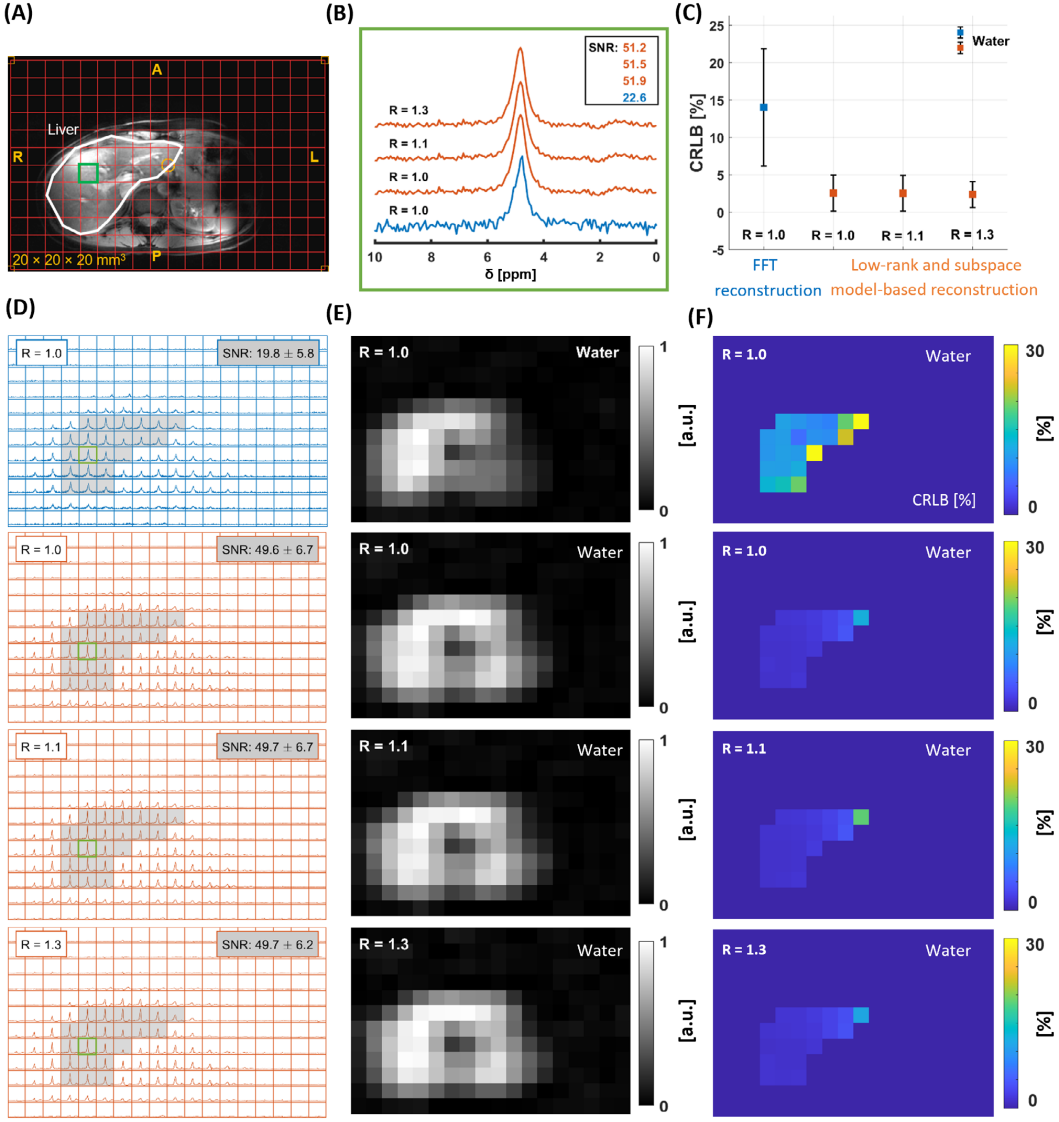
3D deuterium MRSI dataset of the human liver at natural abundance processed by two reconstruction methods: FFT reconstruction and low‐rank and subspace model‐based reconstruction (orange). (A) The ^1^H MRI image (Dixon) shows the liver, contoured with a white line, and the voxel of interest indicated by the green box. (B) Deuterium spectra from this voxel within the liver are shown for both reconstruction methods at different acceleration factors (R = 1.0, 1.1, and 1.3), with corresponding SNR values. The average SNR for the water peak within the liver was computed from the middle slice. (C) CRLB [%] of the fitted water signals was calculated from FFT reconstruction and low‐rank and subspace model‐based reconstruction for R = 1.0, 1.1, and 1.3. (D) Deuterium MRSI spectra from the middle slice are displayed for both reconstruction methods, with the SNR of the water peak calculated across 22 voxels in the liver region (highlighted in gray). Low‐rank and subspace model‐based reconstruction was performed with a D1 size of 3 × 5 × 3 in k‐space and L = 5. (E) Deuterated water metabolite maps are shown for FFT reconstruction (top row) and low‐rank and subspace model‐based reconstruction at R = 1.0, 1.1, and 1.3. (F) CRLB [%] maps of water from both reconstruction methods for the liver ROI are displayed. ^1^H, hydrogen‐1 (proton).

Figure 3 presents the deuterium spectra of the liver at natural abundance levels for both reconstructions. In the liver ROI (Figure 3A), the average SNR (Figure 3D) was approximately 2.6‐fold higher with low‐rank and subspace model‐based reconstruction at R = 1.0, 1.1, and 1.3 compared to FFT reconstruction. A voxel from the middle slice of the 3D ^2^H MRSI was selected, presenting an SNR of approximately 59 in the low‐rank and subspace model‐based reconstruction, 2.6 times higher than FFT (Figure 3B). The CRLB for water in the liver ROI was also calculated (Figure 3C). The CRLB [%] for FFT reconstruction (14.0 ± 7.8) was higher than that for low‐rank and subspace model‐based reconstruction with R = 1.0, 1.1, and 1.3 (2.6 ± 2.4, 2.5 ± 2.4, and 2.4 ± 1.7, respectively). Deuterated water maps (Figure 3E) and CRLB maps (Figure 3F) were consistent across different R for low‐rank reconstruction, with relatively stable and lower CRLB values than FFT reconstruction.

**FIGURE 4:**
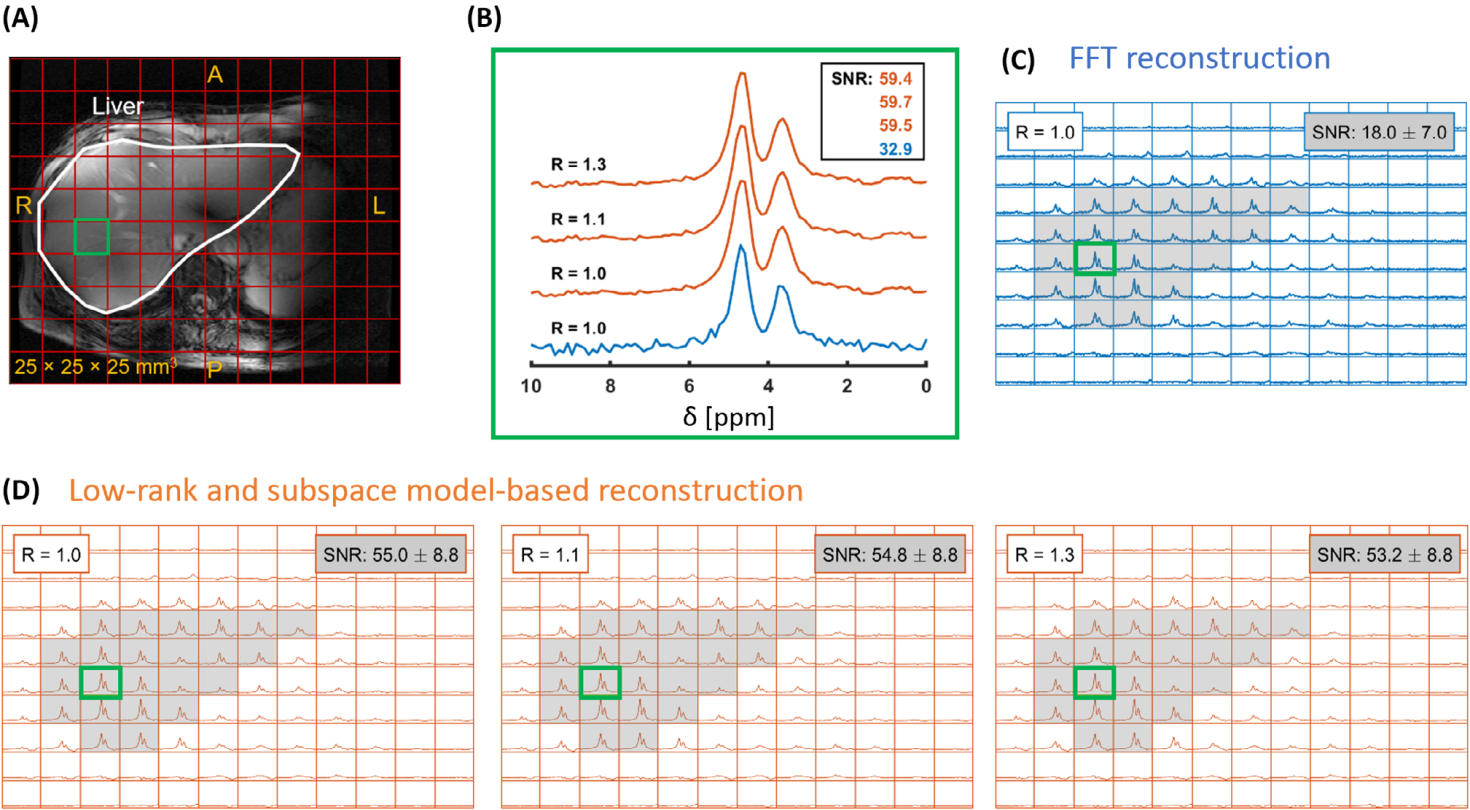
3D DMI datasets captured from the human liver, acquired 2.5 h post‐oral glucose intake, processed using two reconstruction methods: FFT reconstruction (blue) and low‐rank and subspace model‐based reconstruction (orange). (A) The ^1^H MRI image (Dixon) displays the liver, contoured by a white line. The SNR for a selected voxel in the liver ROI (white) was calculated using two reconstruction methods. (B) Water signals from the voxel within the liver (green box) are presented, with SNR values calculated for both FFT reconstruction and low‐rank and subspace model‐based reconstruction at different acceleration factors (R = 1.0, 1.1, and 1.3). (C) FFT reconstruction results are shown for the liver region, with the average SNR of the water peak calculated for 23 voxels in the liver ROI (highlighted in gray). (D) Low‐rank and subspace model‐based reconstruction results are shown for the same 23 voxels in the liver region, with spectra displayed at R = 1.0, 1.1, and 1.3. The SNR is significantly higher than that of FFT reconstruction. Low‐rank and subspace model‐based reconstruction used a D1 size of 3 × 3 × 3 and L = 5.

Figure 4 shows the deuterium spectra acquired 2.5 h after oral glucose intake from both reconstruction methods. For low‐rank and subspace model‐based reconstruction, the SNR was about 1.8 times higher than FFT reconstruction and remained consistent across acceleration factors (Figure 4B). The water SNR in the liver ROI was approximately three times higher for low‐rank and subspace model‐based reconstruction at R = 1.0, 1.1, and 1.3 (Figure 4D) compared to FFT reconstruction (Figure 4C).

**FIGURE 5:**
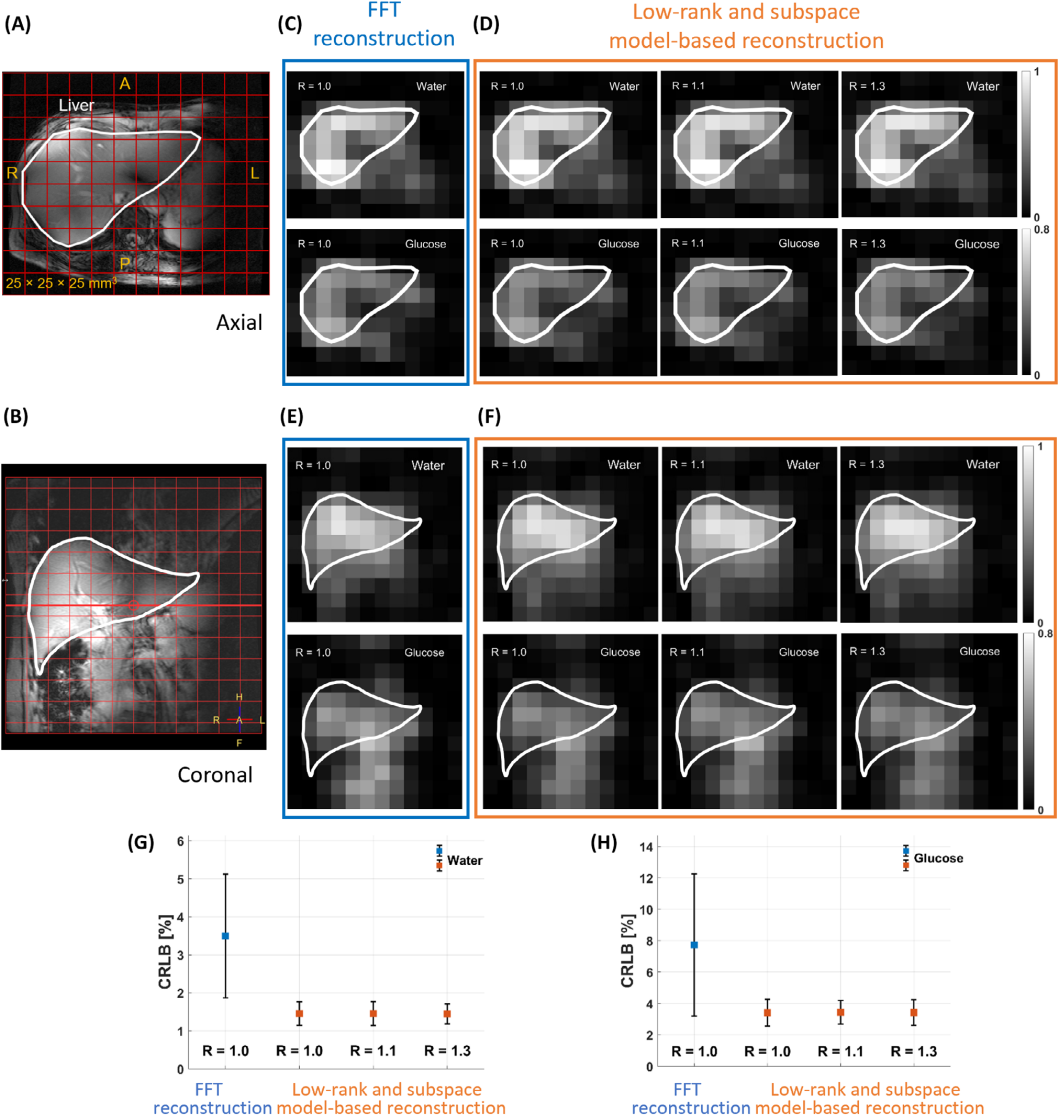
Deuterated water and glucose metabolite maps obtained from DMI data in the axial and coronal planes, processed using FFT reconstruction and low‐rank and subspace model‐based reconstruction. The ^1^H MRI image (Dixon) displays the liver in both axial (A) and coronal (B) planes, outlined with a white contour. The metabolite maps illustrate the intensity of glucose and water signals 2.5 h after oral glucose intake, highlighting the difference in reconstruction methods. Axial deuterated water and glucose maps are shown for both FFT reconstruction (C) and low‐rank and subspace model‐based reconstruction (D) at acceleration factors R = 1.0, 1.1, and 1.3. Similarly, the coronal metabolite maps are displayed, again comparing FFT reconstruction (E) and low‐rank and subspace model‐based reconstruction (F) at the same acceleration factors. Both methods show similar patterns in the metabolite distribution, but the low‐rank method consistently provides still higher signal intensities and good definition, even at higher acceleration factor. The CRLB [%] of the deuterated water (G) and glucose (H) signals were calculated for both reconstruction methods, with the FFT reconstruction exhibiting higher CRLB values compared to the low‐rank and subspace model‐based reconstruction across all acceleration factors. This indicates lower fitting accuracy in FFT reconstruction compared to the low‐rank approach, particularly for water and glucose signals. DMI, deuterium metabolic imaging.

Figure 5 displays water and glucose maps from both reconstructions. The maps from FFT reconstruction (Figure 5C,E) in the transverse and coronal planes (Figure 5A,B) appeared visually similar to those from low‐rank and subspace model‐based reconstruction (Figure 5D,F). However, CRLB for water and glucose was about 2.3 times higher with FFT reconstruction than with low‐rank and subspace model‐based reconstruction for both metabolites (Figure 5G,H).

## 5 DISCUSSION

We successfully implemented the low‐rank and subspace model‐based reconstruction to evaluate its performance on 3D DMI data acquired from the human liver at 7 T, using both fully sampled and undersampled data. The method's performance was assessed through simulations and in vivo experiments, both at natural abundance deuterium levels and after oral intake of deuterated glucose. Comparative analysis of simulations and in vivo experiments with FFT reconstruction showed that the low‐rank and subspace model‐based reconstruction reduced noise, leading to higher SNR and significantly lower CRLB and NRMSE.

The reconstructed metabolite maps in the simulation showed significant similarity to the reference ground truth. Compared to FFT reconstruction, the CRLB of signals from low‐rank and subspace model‐based reconstruction was significantly lower, indicating improved parameter estimation accuracy. Moreover, the overall NRMSE indicated that the inaccuracy of fitted signals from low‐rank and subspace model‐based reconstruction was also lower than that of FFT reconstruction; the improvement in NRMSE was more modest. For water and glucose, NRMSE remained lower with low‐rank and subspace model‐based reconstruction even at R = 1.3, although it was higher for Glx and lipid. However, below R = 1.3, low‐rank and subspace model‐based reconstruction consistently had lower NRMSE for all metabolites, demonstrating its superior performance over FFT reconstruction.

When comparing metabolite maps from both reconstructions to the ground truth, FFT reconstruction produced more uniformly distributed mean bias maps than low‐rank and subspace model‐based reconstruction. However, maps generated using temporal bases from the ground truth in low‐rank, and subspace model‐based reconstruction showed a more uniform distribution than those using noisy data. Despite this, FFT reconstruction had higher mean bias SD across all metabolites. In contrast, low‐rank and subspace model‐based reconstruction produced more uniform and lower mean bias SD maps. Thus, even with acceleration, estimating temporal bases from the ground truth may result in more uniformly distributed mean bias maps.

In our in vivo experiments, the Glx signal was not detected despite the SNR being sufficiently high (˜60). Lipid signals were observed at the liver boundary in the second volunteer with both reconstruction methods (Figure S3). However, the chemical shift range of lipid (0.9–1.3 ppm) overlaps with that of lactate, making differentiation between them challenging. Because this study involved a healthy volunteer, lactate was not clearly detected in the liver, reducing potential confusion in interpreting the lipid signals. Water and glucose maps, in contrast, were similar across both FFT and low‐rank and subspace model‐based reconstructions, even at higher acceleration factors (Figure S4). Future studies could benefit from methods such as spectral localization by imaging (SLIM)^67^ or the union‐of‐subspaces model,^68^ which suppress lipid signals and enhance the detection of metabolic signals within the liver.

### 5.1 Current limitations

In the simulation study, we observed the lowest mean bias when the temporal basis was estimated from noise‐free data (Figure 2D). However, in our in vivo DMI data, the temporal basis was estimated from the k‐space of noisy data in the low‐rank and subspace model‐based reconstruction. When using quantum mechanical simulations to predict the resonance structures of individual metabolites and specially designed trained data to learn the distribution of molecular‐dependent parameters, experiment‐dependent limited central k‐space might be no longer needed for subspace estimation.^69^

The current acceleration factor of 1.3, demonstrated using retrospective undersampling on data obtained with a Hamming‐weighted acquisition, provides a moderate improvement. This factor could be enhanced in the future with the implementation of rapid acquisition sequences, higher‐resolution data, and a greater number of receiver coils, enabling more substantial acceleration without compromising data quality.

Respiratory motion is known to degrade MRS spectra by causing B0 field fluctuations, leading to broadened spectral lines, frequency shifts, and signal loss.^70^ Although motion correction was not applied in our study, the metabolic data quality was sufficient for analysis. Future work may benefit from incorporating motion correction techniques (e.g., FID navigators),^70^, ^71^ which may reduce ghosting and enhance SNR by narrowing linewidths for metabolites near the liver.

## 6 CONCLUSION

This work demonstrates the application of low‐rank subspace model‐based reconstruction for 3D DMI data at 7 T. This reconstruction method improves SNR, reduces NRMSE and CRLB, and enhances metabolite detection. Both simulation and in vivo experiments showed that acquisition times could be shortened without compromising spatial resolution or spectral quality. Such improvements could be particularly advantageous in clinical settings where time constraints are critical, potentially enhancing the practicality of 3D high‐resolution DMI.

## FUNDING INFORMATION

This project has received funding from the European Union's Horizon 2020 research and innovation program under the Marie Sklodowska‐Curie grant agreement (813120), MITI (EU EIC Transition) Non‐ionizing Metabolic Imaging for predicting the effect of and guiding Therapeutic Interventions (101058229), and supported by ITEA4 under the Spectralligence project (20209).

## Supporting information


**Figure S1.** NRMSE as a function of regularization parameters (λ) for different metabolites in low‐rank and subspace model‐based reconstruction at R = 1.3. (A) NRMSE for water shows relatively consistent values across different λ, with only slight variations as the regularization increases. (B) NRMSE for glucose remains stable at lower λ values but increases significantly at λ = 0.03 and 0.05, indicating higher error with larger regularization. (C) NRMSE for Glx remains stable at lower λ values. (D) NRMSE for lipid shows a notable increase in error as λ increases, particularly for λ = 0.03 and 0.05, indicating more sensitivity to regularization.
**Figure S2.** Plot of singular values versus model order (rank L). The normalized singular value spectrum of the Casorati matrix was calculated from the simulation study using noisy data with SNR of 15. After the model order reaches 5, the singular values approach zero.
**Figure S3.** Lipid maps of the human liver obtained from 3D DMI, processed using two reconstruction methods. (A) Axial view and (B) coronal view of the liver from a ^1^H MRI image (Dixon), with the liver outlined in white. The grid shows the voxel size (25 × 25 × 25 mm^3^). (C, E) Lipid maps reconstructed using FFT reconstruction (R = 1.0) in the axial and coronal planes, respectively. (D, F) Lipid maps reconstructed using the low‐rank and subspace model‐based reconstruction at acceleration factors of R = 1.0, 1.1, and 1.3 in both axial and coronal planes. The intensity range is consistent across lipid maps with the color bar indicating signal intensity from 0 to 0.3 [a.u].
**Figure S4.** Deuterated water, glucose, and lipid metabolite maps derived from DMI data in axial and coronal planes, processed using FFT and low‐rank and subspace model‐based reconstructions. The ^1^H Dixon MRI images show the liver in axial (A) and coronal (B) views, outlined with a white contour. The metabolite maps present the intensity of glucose and water signals 2.5 h post oral glucose intake, emphasizing differences between the reconstruction methods. Axial deuterated water, glucose, and lipid maps are displayed for FFT reconstruction (C) and low‐rank and subspace model‐based reconstruction (D) at acceleration factors R = 1.0 and 2.0. Similarly, coronal plane metabolite maps are shown for FFT reconstruction (E) and low‐rank and subspace model‐based reconstructions (F) at the same acceleration factors. Both methods demonstrate similar distribution patterns for water and glucose maps, even at R = 2.0. While water and glucose maps remain similar at R = 2.0 for both methods, noticeable differences appear in the lipid map when comparing R = 1.0 and R = 2.0 in the low‐rank and subspace model‐based reconstruction, with lipid signals becoming more variable at higher acceleration. The CRLB [%] values for deuterated water (G) and glucose (H) were calculated for both methods, with FFT reconstruction showing higher CRLB values across all acceleration factors, indicating reduced fitting accuracy compared to the low‐rank approach, particularly for water and glucose signals.
**Table S1.** Scan parameters for the MR measurements at natural abundance deuterium levels (2nd column) and for deuterated glucose intake (3rd column).

## Data Availability

The codes and simulation data (in the data format used by the BART toolbox) that support the findings of this study are openly available on GitHub at https://github.com/Kyungmin‐Nam/DMI_LowRank_and_Subspace_Recon.

## References

[1] De Feyter HM , Behar KL , Corbin ZA , et al. Deuterium metabolic imaging (DMI) for MRI‐based 3D mapping of metabolism in vivo. Sci Adv. 2018;4:eaat7314. 30140744 10.1126/sciadv.aat7314 PMC6105304

[2] De Feyter HM , de Graaf RA . Deuterium metabolic imaging – back to the future. J Magn Reson. 2021;326:106932. 33902815 10.1016/j.jmr.2021.106932 PMC8083995

[3] Straathof M , Meerwaldt AE , De Feyter HM , de Graaf RA , Dijkhuizen RM . Deuterium metabolic imaging of the healthy and diseased brain. Neuroscience. 2021;474:94‐99. 33493618 10.1016/j.neuroscience.2021.01.023 PMC9846473

[4] Ip KL , Thomas MA , Behar KL , de Graaf RA , De Feyter HM . Mapping of exogenous choline uptake and metabolism in rat glioblastoma using deuterium metabolic imaging (DMI). Front Cell Neurosci. 2023;17:1130816. 37187610 10.3389/fncel.2023.1130816 PMC10175635

[5] Warburg VO . Über den Stoffwechsel der Carcinomzelle. Naturwissenschaften. 1924;12:1131‐1137.

[6] Koppenol WH , Bounds PL , Dang CV . Otto Warburg's contributions to current concepts of cancer metabolism. Nat Rev Cancer. 2011;11:325‐337. 21508971 10.1038/nrc3038

[7] Bogner W , Otazo R , Henning A . Accelerated MR spectroscopic imaging—a review of current and emerging techniques. NMR Biomed. 2021;34(5):e4314. 32399974 10.1002/nbm.4314 PMC8244067

[8] Vidya Shankar R , Chang JC , Hu HH , Kodibagkar VD . Fast data acquisition techniques in magnetic resonance spectroscopic imaging. NMR Biomed. 2019;32(3):e4046. 30637822 10.1002/nbm.4046

[9] Posse S , Tedeschi G , Risinger R , Ogg R , Le BD . High speed 1H spectroscopic imaging in human brain. Magn Reson Med. 1995;33:34‐40. 7891533 10.1002/mrm.1910330106

[10] Nam KM , Gursan A , Bhogal AA , et al. Deuterium echo‐planar spectroscopic imaging (EPSI) in the human liver in vivo at 7 T. Magn Reson Med. 2023;90:863‐874. 37154391 10.1002/mrm.29696

[11] Ebel A , Maudsley AA , Weiner MW , Schuff N . Achieving sufficient spectral bandwidth for volumetric 1H echo‐planar spectroscopic imaging at 4 Tesla. Magn Reson Med. 2005;54:697‐701. 16086316 10.1002/mrm.20593 PMC1851680

[12] Chatnuntawech I , Gagoski B , Bilgic B , Cauley SF , Setsompop K , Adalsteinsson E . Accelerated 1H MRSI using randomly undersampled spiral‐based k‐space trajectories. Magn Reson Med. 2015;74:13‐24. 25079076 10.1002/mrm.25394

[13] Adalsteinsson E , Irarrazabal P , Topp S , Meyer C , Macovski A , Spielman DM . Volumetric spectroscopic imaging with spiral‐based k‐space trajectories. Magn Reson Med. 1998;39:889‐898. 9621912 10.1002/mrm.1910390606

[14] Furuyama JK , Wilson NE , Thomas MA . Spectroscopic imaging using concentrically circular echo‐planar trajectories in vivo. Magn Reson Med. 2012;67:1515‐1522. 22006586 10.1002/mrm.23184

[15] Chiew M , Jiang W , Burns B , et al. Density‐weighted concentric rings k‐space trajectory for 1H magnetic resonance spectroscopic imaging at 7 T. NMR Biomed. 2018;31(1):e3838. 29044762 10.1002/nbm.3838 PMC5969060

[16] Hingerl L , Bogner W , Moser P , et al. Density‐weighted concentric circle trajectories for high resolution brain magnetic resonance spectroscopic imaging at 7T. Magn Reson Med. 2018;79:2874‐2885. 29106742 10.1002/mrm.26987 PMC5873433

[17] Ramirez MS , Lee J , Walker CM , et al. Radial spectroscopic MRI of hyperpolarized [1‐13C] pyruvate at 7 Tesla. Magn Reson Med. 2014;72:986‐995. 24186845 10.1002/mrm.25004 PMC4007384

[18] Schirda CV , Tanase C , Boada FE . Rosette spectroscopic imaging: optimal parameters for alias‐free, high sensitivity spectroscopic imaging. J Magn Reson Imaging. 2009;29:1375‐1385. 19472411 10.1002/jmri.21760

[19] Nassirpour S , Chang P , Avdievitch N , Henning A . Compressed sensing for high‐resolution nonlipid suppressed 1H FID MRSI of the human brain at 9.4T. Magn Reson Med. 2018;80:2311‐2325. 29707804 10.1002/mrm.27225

[20] Larson PEZ , Hu S , Lustig M , et al. Fast dynamic 3D MR spectroscopic imaging with compressed sensing and multiband excitation pulses for hyperpolarized 13C studies. Magn Reson Med. 2011;65:610‐619. 20939089 10.1002/mrm.22650 PMC3021589

[21] Korzowski A , Bachert P . High‐resolution 31P echo‐planar spectroscopic imaging in vivo at 7T. Magn Reson Med. 2018;79:1251‐1259. 28639310 10.1002/mrm.26785

[22] Dydak U , Weiger M , Pruessmann KP , Meier D , Boesiger P . Sensitivity‐encoded spectroscopic imaging. Magn Reson Med. 2001;46:713‐722. 11590648 10.1002/mrm.1250

[23] Kirchner T , Fillmer A , Tsao J , Pruessmann KP , Henning A . Reduction of voxel bleeding in highly accelerated parallel 1H MRSI by direct control of the spatial response function. Magn Reson Med. 2015;73:469‐480. 24585512 10.1002/mrm.25185

[24] Strasser B , Považan M , Hangel G , et al. (2 + 1)D‐CAIPIRINHA accelerated MR spectroscopic imaging of the brain at 7T. Magn Reson Med. 2017;78:429‐440. 27548836 10.1002/mrm.26386 PMC5535010

[25] Lin FH , Tsai SY , Otazo R , et al. Sensitivity‐encoded (SENSE) proton echo‐planar spectroscopic imaging (PEPSI) in the human brain. Magn Reson Med. 2007;57:249‐257. 17260356 10.1002/mrm.21119

[26] Froeling M , Prompers JJ , Klomp DWJ , van der Velden TA . PCA denoising and Wiener deconvolution of 31P 3D CSI data to enhance effective SNR and improve point spread function. Magn Reson Med. 2021;85:2992‐3009. 33522635 10.1002/mrm.28654 PMC7986807

[27] Clarke WT , Chiew M . Uncertainty in denoising of MRSI using low‐rank methods. Magn Reson Med. 2022;87:574‐588. 34545962 10.1002/mrm.29018 PMC7612041

[28] Abdoli A , Stoyanova R , Maudsley AA . Denoising of MR spectroscopic imaging data using statistical selection of principal components. MAGMA. 2016;29:811‐822. 27260664 10.1007/s10334-016-0566-z PMC5699222

[29] Nguyen HM , Peng X , Do MN , Liang ZP . Denoising MR spectroscopic imaging data with low‐rank approximations. IEEE Trans Biomed Eng. 2013;60:78‐89. 23070291 10.1109/TBME.2012.2223466 PMC3800688

[30] Nguyen HM , Peng X , Do MN , Liang ZP . Spatiotemporal denoising of MR spectroscopic imaging data by low‐rank approximations. In Proceedings of the 2011 IEEE International Symposium on Biomedical Imaging (ISBI): From Nano to Macro, Chicago, IL. 2011;857‐860. https://ieeexplore.ieee.org/document/5872539.

[31] Nam KM , Hendriks AD , Boer VO , Klomp DWJ , Wijnen JP , Bhogal A . Proton metabolic mapping of the brain at 7 T using a two‐dimensional free induction decay–echo‐planar spectroscopic imaging readout with lipid suppression. NMR Biomed. 2022;35(10):e4771. 35577344 10.1002/nbm.4771 PMC9541868

[32] Mansfield P . Spatial mapping of the chemical shift in NMR. Magn Reson Med. 1984;1:370‐386. 6571566 10.1002/mrm.1910010308

[33] Feinberg DA , Turner R , Jakab PD , von Kienlin M . Echo‐planar imaging with asymmetric gradient modulation and inner‐volume excitation. Magn Reson Med. 1990;13:162‐169. 2319932 10.1002/mrm.1910130116

[34] Wilhelm T , Bachert P . In vivo 31 P echo‐planar spectroscopic imaging of human calf muscle. J Magn Reson. 2001;149:126‐130. 11273761 10.1006/jmre.2001.2288

[35] Ulrich M , Wokrina T , Ende G , Lang M , Bachert P . 31P‐{1H} echo‐planar spectroscopic imaging of the human brain in vivo. Magn Reson Med. 2007;57:784‐790. 17390361 10.1002/mrm.21192

[36] Cunningham CH , Chen AP , Albers MJ , et al. Double spin‐echo sequence for rapid spectroscopic imaging of hyperpolarized 13C. J Magn Reson. 2007;187:357‐362. 17562376 10.1016/j.jmr.2007.05.014

[37] Yen YF , Kohler SJ , Chen AP , et al. Imaging considerations for in vivo 13C metabolic mapping using hyperpolarized 13C‐pyruvate. Magn Reson Med. 2009;62:1‐10. 19319902 10.1002/mrm.21987 PMC2782538

[38] Lam F , Liang ZP . A subspace approach to high‐resolution spectroscopic imaging. Magn Reson Med. 2014;71:1349‐1357. 24496655 10.1002/mrm.25168 PMC4051394

[39] Lam F , Ma C , Clifford B , Johnson CL , Liang ZP . High‐resolution 1H‐MRSI of the brain using SPICE: data acquisition and image reconstruction. Magn Reson Med. 2016;76:1059‐1070. 26509928 10.1002/mrm.26019 PMC4848237

[40] Ma C , Clifford B , Liu Y , et al. High‐resolution dynamic 31P‐MRSI using a low‐rank tensor model. Magn Reson Med. 2017;78:419‐428. 28556373 10.1002/mrm.26762 PMC5562044

[41] Lee H , Song JE , Shin J , et al. High resolution hyperpolarized 13C MRSI using SPICE at 9.4T. Magn Reson Med. 2018;80:703‐710. 29315780 10.1002/mrm.27061

[42] Lam F , Chu J , Choi JS , et al. Epigenetic MRI: noninvasive imaging of DNA methylation in the brain. Proc Natl Acad Sci U S A. 2022;119:e2119891119. 35235458 10.1073/pnas.2119891119 PMC8915962

[43] Guo R , Zhao Y , Li Y , Li Y , Liang ZP . Simultaneous metabolic and functional imaging of the brain using SPICE. Magn Reson Med. 2019;82:1993‐2002. 31294487 10.1002/mrm.27865 PMC6717045

[44] Guo R , Zhao Y , Li Y , et al. Simultaneous QSM and metabolic imaging of the brain using SPICE: further improvements in data acquisition and processing. Magn Reson Med. 2021;85:970‐977. 32810319 10.1002/mrm.28459 PMC7722130

[45] Liang Z‐P . Spatiotemporal imaging with partially separable functions. In Proceedings of the 2007 4th IEEE International Symposium On Biomedical Imaging (ISBI): From Nano to Macro, Arlington, VA; IEEE; 2007:988‐991.

[46] Zhao B , Lu W , Hitchens TK , Lam F , Ho C , Liang ZP . Accelerated MR parameter mapping with low‐rank and sparsity constraints. Magn Reson Med. 2015;74:489‐498. 25163720 10.1002/mrm.25421 PMC4344441

[47] Zhao B , Setsompop K , Adalsteinsson E , et al. Improved magnetic resonance fingerprinting reconstruction with low‐rank and subspace modeling. Magn Reson Med. 2018;79:933‐942. 28411394 10.1002/mrm.26701 PMC5641478

[48] Li Y , Lam F , Clifford B , Liang ZP . A subspace approach to spectral quantification for MR spectroscopic imaging. IEEE Trans Biomed Eng. 2017;64:2486‐2489. 28829303 10.1109/TBME.2017.2741922 PMC5646283

[49] Zhu X‐H , Wiesner MH , Li Y , et al. Differentiating TCA cycle activity of gray and white matter in human brain at 7T using high resolution dynamic deuterium MRS imaging with SPICE. Proceedings of the 30th Joint Annual ISMRM‐ESMRMB Meeting; 2022: Abstract #4840.

[50] Han PK , Ma C , Deng K , et al. A minimum‐phase Shinnar‐Le Roux spectral‐spatial excitation RF pulse for simultaneous water and lipid suppression in 1H‐MRSI of body extremities. Magn Reson Imaging. 2018;45:18‐25. 28917812 10.1016/j.mri.2017.09.008 PMC5709164

[51] Wiesner HM , Guo R , Li Y , et al. High‐resolution 3D phosphorus metabolic imaging of the human brain at 7T using SPICE. In Proceedings of the 29th Annual Meeting of the ISMRM, Virtual Conference, 2021. p. 0235.

[52] Zhao Y , Hitchens TK , Herneisey M , et al. Fast high‐resolution 19F‐MRSI of perfluorocarbon Nanoemulsions for MRI cell tracking using SPICE with learned subspaces. Proceedings of the 29th Annual Meeting of ISMRM & SMRT Virtual Conference & Exhibition; 2021: Abstract #0341.

[53] Song JE , Shin J , Lee H , Choi YS , Song HT , Kim DH . Dynamic hyperpolarized 13C MR spectroscopic imaging using SPICE in mouse kidney at 9.4 T. NMR Biomed. 2020;33:e4230. 31856426 10.1002/nbm.4230

[54] Clifford B , Gu Y , Liu Y , et al. High‐resolution dynamic 31P‐MR spectroscopic imaging for mapping mitochondrial function. IEEE Trans Biomed Eng. 2020;67:2745‐2753. 32011244 10.1109/TBME.2020.2969892 PMC7384926

[55] Zhu X‐H , Li X , Li Y , et al. Evaluating the clinical utility and diagnostic value of high‐resolution deuterium MRS imaging (DMRSI) in patients with brain tumor. Proceedings of the 32nd Annual Joint ISMRM & ISMRT Meeting; 2024: Abstract #0363.

[56] Chambolle A , Pock T . A first‐order primal‐dual algorithm for convex problems with applications to imaging. J Math Imaging Vis. 2011;40:120‐145.

[57] Uecker M , Tamir JI , Ong F , Lustig M . The BART toolbox for computational magnetic resonance imaging. In Proceedings of the 24th Annual Meeting of ISMRM, Singapore, 2016. doi: 10.5281/zenodo.592960

[58] Tamir JI , Uecker M , Chen W , et al. T2 shuffling: sharp, multicontrast, volumetric fast spin‐echo imaging. Magn Reson Med. 2017;77:180‐195. 26786745 10.1002/mrm.26102 PMC4990508

[59] Gursan A , Hendriks A , Welting D , de Jong P , Klomp D , Prompers J . Deuterium body array for the simultaneous measurement of hepatic and renal glucose metabolism and gastric emptying with dynamic 3D deuterium metabolic imaging at 7T. NMR Biomed. 2023;36:e4926. 36929629 10.1002/nbm.4926 PMC13587257

[60] Schär M , Kozerke S , Fischer SE , Boesiger P . Cardiac SSFP imaging at 3 Tesla. Magn Reson Med. 2004;51:799‐806. 15065254 10.1002/mrm.20024

[61] Uecker M , Lai P , Murphy MJ , et al. ESPIRiT ‐ an eigenvalue approach to autocalibrating parallel MRI: where SENSE meets GRAPPA. Magn Reson Med. 2014;71:990‐1001. 23649942 10.1002/mrm.24751 PMC4142121

[62] Segars WP , Sturgeon G , Mendonca S , Grimes J , Tsui BMW . 4D XCAT phantom for multimodality imaging research. Med Phys. 2010;37:4902‐4915. 20964209 10.1118/1.3480985 PMC2941518

[63] Paganelli C , Summers P , Gianoli C , Bellomi M , Baroni G , Riboldi M . A tool for validating MRI‐guided strategies: a digital breathing CT/MRI phantom of the abdominal site. Med Biol Eng Comput. 2017;55:2001‐2014. 28390002 10.1007/s11517-017-1646-6

[64] Juchem C , Cudalbu C , de Graaf RA , et al. B0 shimming for in vivo magnetic resonance spectroscopy: experts' consensus recommendations. NMR Biomed. 2021;34:e4350. 32596978 10.1002/nbm.4350

[65] Vanhamme L , Van Den Boogaart A , Van Huffel S . Improved method for accurate and efficient quantification of MRS data with use of prior knowledge. J Magn Reson. 1997;129:35‐43. 9405214 10.1006/jmre.1997.1244

[66] Purvis LAB , Clarke WT , Biasiolli L , Valkovič L , Robson MD , Rodgers CT . OXSA: an open‐source magnetic resonance spectroscopy analysis toolbox in MATLAB. PLoS One. 2017;12(9):e0185356. 28938003 10.1371/journal.pone.0185356 PMC5609763

[67] De Graaf RA , Liu Y , Corbin ZA , De Feyter HM . Lipid removal in deuterium metabolic imaging (DMI) using spatial prior knowledge. Magn Reson Discuss. 2024;5:21‐31. 10.5194/mr-5-21-2024 PMC11836565 39980872

[68] Ma C , Lam F , Johnson CL , Liang ZP . Removal of nuisance signals from limited and sparse 1H MRSI data using a union‐of‐subspaces model. Magn Reson Med. 2016;75:488‐497. 25762370 10.1002/mrm.25635 PMC4567537

[69] Lam F , Li Y , Guo R , Clifford B , Liang ZP . Ultrafast magnetic resonance spectroscopic imaging using SPICE with learned subspaces. Magn Reson Med. 2020;83:377‐390. 31483526 10.1002/mrm.27980 PMC6824949

[70] Andronesi OC , Bhattacharyya PK , Bogner W , et al. Motion correction methods for MRS: experts' consensus recommendations. NMR Biomed. 2021;34(5):e4364. 33089547 10.1002/nbm.4364 PMC7855523

[71] Wallace TE , Afacan O , Waszak M , Kober T , Warfield SK . Head motion measurement and correction using FID navigators. Magn Reson Med. 2019;81:258‐274. 30058216 10.1002/mrm.27381 PMC6258267

