# Supplementary material for: 3D deuterium metabolic imaging (DMI) of the human liver at 7 T using low‐rank and subspace model‐based reconstruction
Source: Magn Reson Med. 2024 Dec 22;93(5):1860–73. doi: 10.1002/mrm.30395 (PMC11893041; doi:10.1002/mrm.30395)
Supplement: Supplementary file 1 — Figure S1. NRMSE as a function of regularization parameters (λ) for different metabolites in low‐rank and subspace model‐based reconstruction at R = 1.3. (A) NRMSE for water shows relatively consistent values across different λ, with only slight variations as the regularization increases. (B) NRMSE for glucose remains stable at lower λ values but increases significantly at λ = 0.03 and 0.05, indicating higher error with larger regularization. (C) NRMSE for Glx remains stable at lower λ values. (D) NRMSE for lipid shows a notable increase in error as λ increases, particularly for λ = 0.03 and 0.05, indicating more sensitivity to regularization. Figure S2. Plot of singular values versus model order (rank L). The normalized singular value spectrum of the Casorati matrix was calculated from the simulation study using noisy data with SNR of 15. After the model order reaches 5, the singular values approach zero. Figure S3. Lipid maps of the human liver obtained from 3D DMI, processed using two reconstruction methods. (A) Axial view and (B) coronal view of the liver from a 1H MRI image (Dixon), with the liver outlined in white. The grid shows the voxel size (25 × 25 × 25 mm3). (C, E) Lipid maps reconstructed using FFT reconstruction (R = 1.0) in the axial and coronal planes, respectively. (D, F) Lipid maps reconstructed using the low‐rank and subspace model‐based reconstruction at acceleration factors of R = 1.0, 1.1, and 1.3 in both axial and coronal planes. The intensity range is consistent across lipid maps with the color bar indicating signal intensity from 0 to 0.3 [a.u]. Figure S4. Deuterated water, glucose, and lipid metabolite maps derived from DMI data in axial and coronal planes, processed using FFT and low‐rank and subspace model‐based reconstructions. The 1H Dixon MRI images show the liver in axial (A) and coronal (B) views, outlined with a white contour. The metabolite maps present the intensity of glucose and water signals 2.5 h post oral glucose intake, emph [file MRM-93-1860-s001.docx]

**Supporting information document**

| Scan parameters | Natural Abundance | Deuterated Glucose Intake |
| --- | --- | --- |
| Field of View (FOV) | 240 (AP) × 360 (RL) × 300 (FH) mm^3^ | 250 (AP) × 300 (RL) × 300 (FH) mm^3^ |
| Voxel Size | 20 × 20 × 20 mm^3^ | 25 × 25 × 25 mm^3^ |
| Repetition Time (TR) | 371 ms | 333 ms |
| Echo Time (TE) | 1.0 ms | 1.95 ms |
| RF pulse duration | 1 ms | 1 ms |
| Spectral Bandwidth | 1443 Hz | 5000 Hz |
| Number of Time Samples | 512 | 1024 |
| Weighted Averages | 4 | 4 |
| Acquisition Time | 25 min 56 s | 10 min 35 s |

**Table S1:** Scan parameters for the MR measurements at natural abundance deuterium levels (2^nd^ column) and for deuterated glucose intake (3^rd^ column).


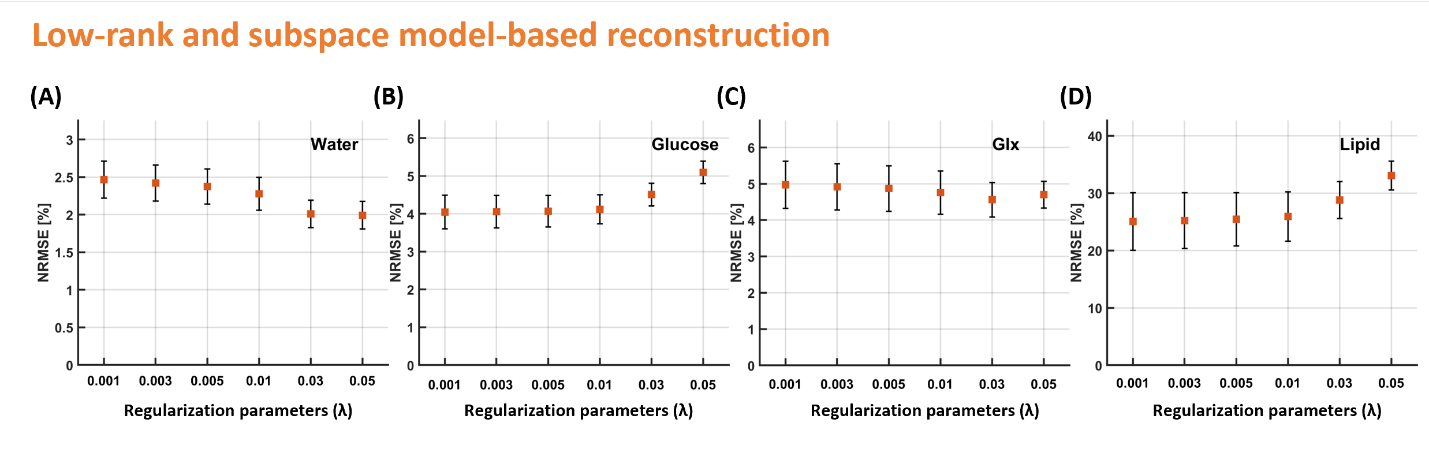


**Figure S1:** NRMSE as a function of regularization parameters (λ) for different metabolites in low-rank and subspace model-based reconstruction at R = 1.3. **(A)** NRMSE for water shows relatively consistent values across different λ, with only slight variations as the regularization increases. **(B)** NRMSE for glucose remains stable at lower λ values but increases significantly at λ = 0.03 and 0.05, indicating higher error with larger regularization. **(C)** NRMSE for Glx remains stable at lower λ values. **(D)** NRMSE for lipid shows a notable increase in error as λ increases, particularly for λ = 0.03 and 0.05, indicating more sensitivity to regularization.


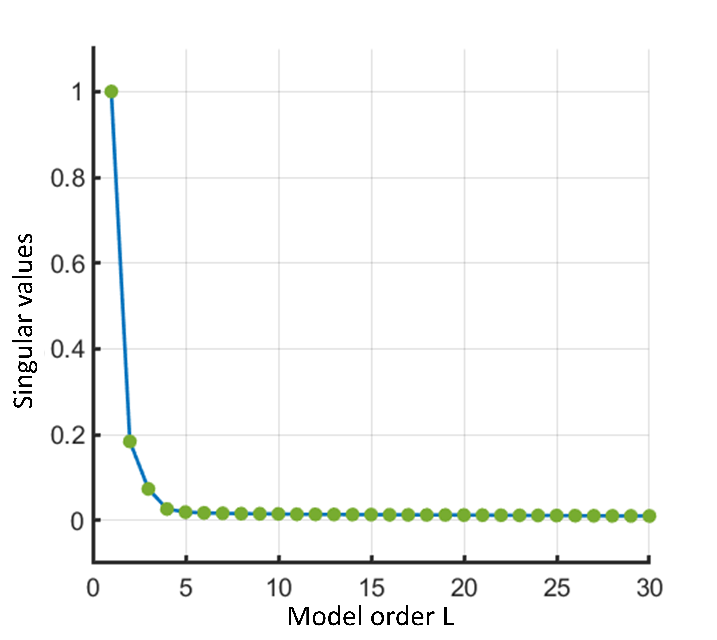
**Figure S2:** Plot of singular values versus model order (L). The normalized singular value spectrum of the Casorati matrix was calculated from the simulation study using noisy data with SNR of 15. After the model order reaches 5, the singular values approach zero.


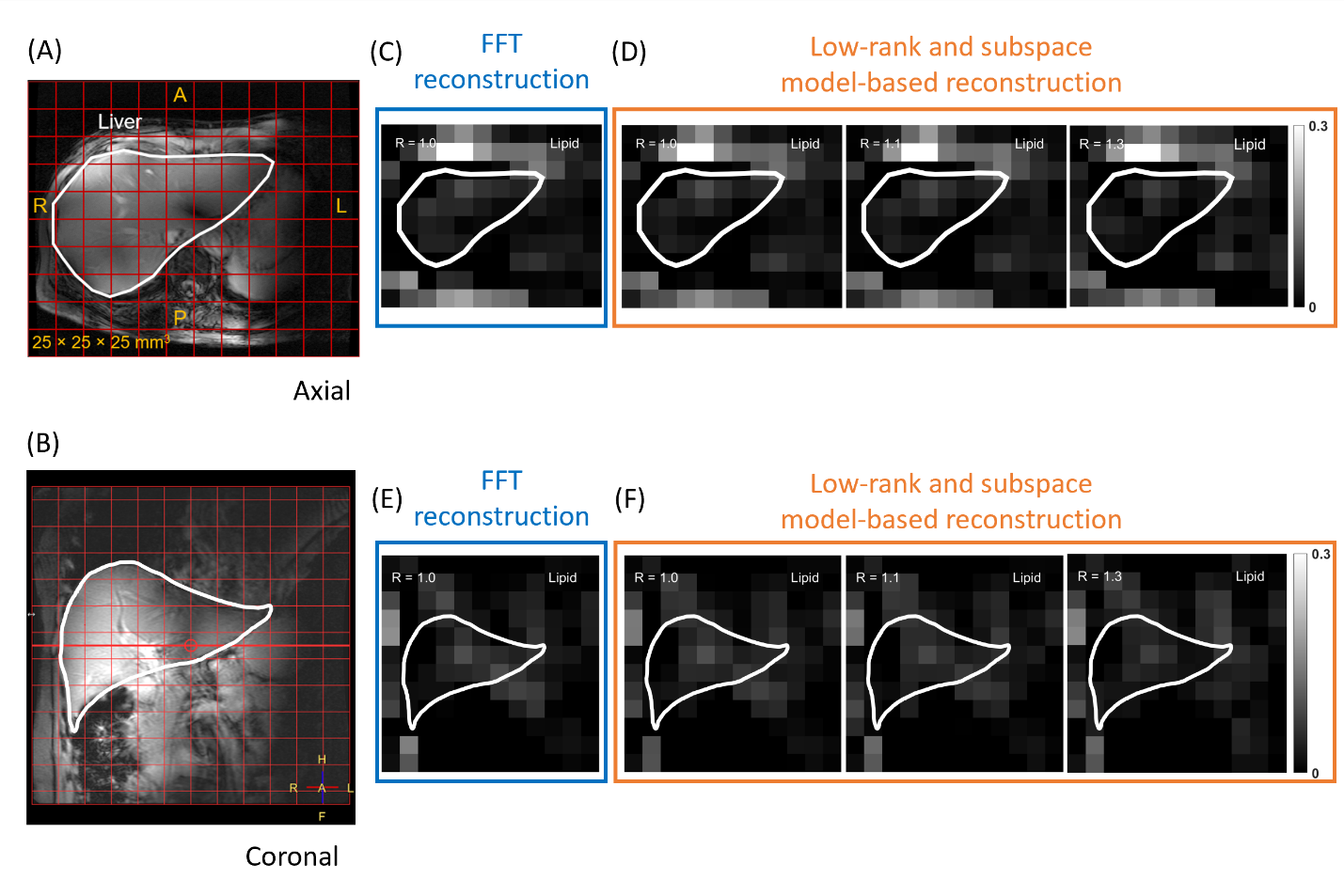
**Figure S3:** Lipid maps of the human liver obtained from 3D DMI, processed using two reconstruction methods. **(A)** Axial view and **(B)** coronal view of the liver from a ¹H MRI image (Dixon), with the liver outlined in white. The grid shows the voxel size (25 × 25 × 25 mm³). **(C, E)** Lipid maps reconstructed using FFT reconstruction (R = 1.0) in the axial and coronal planes, respectively. **(D, F)** Lipid maps reconstructed using the low-rank and subspace model-based reconstruction at acceleration factors of R = 1.0, 1.1, and 1.3 in both axial and coronal planes. The intensity range is consistent across lipid maps with the color bar indicating signal intensity from 0 to 0.3 [a.u].


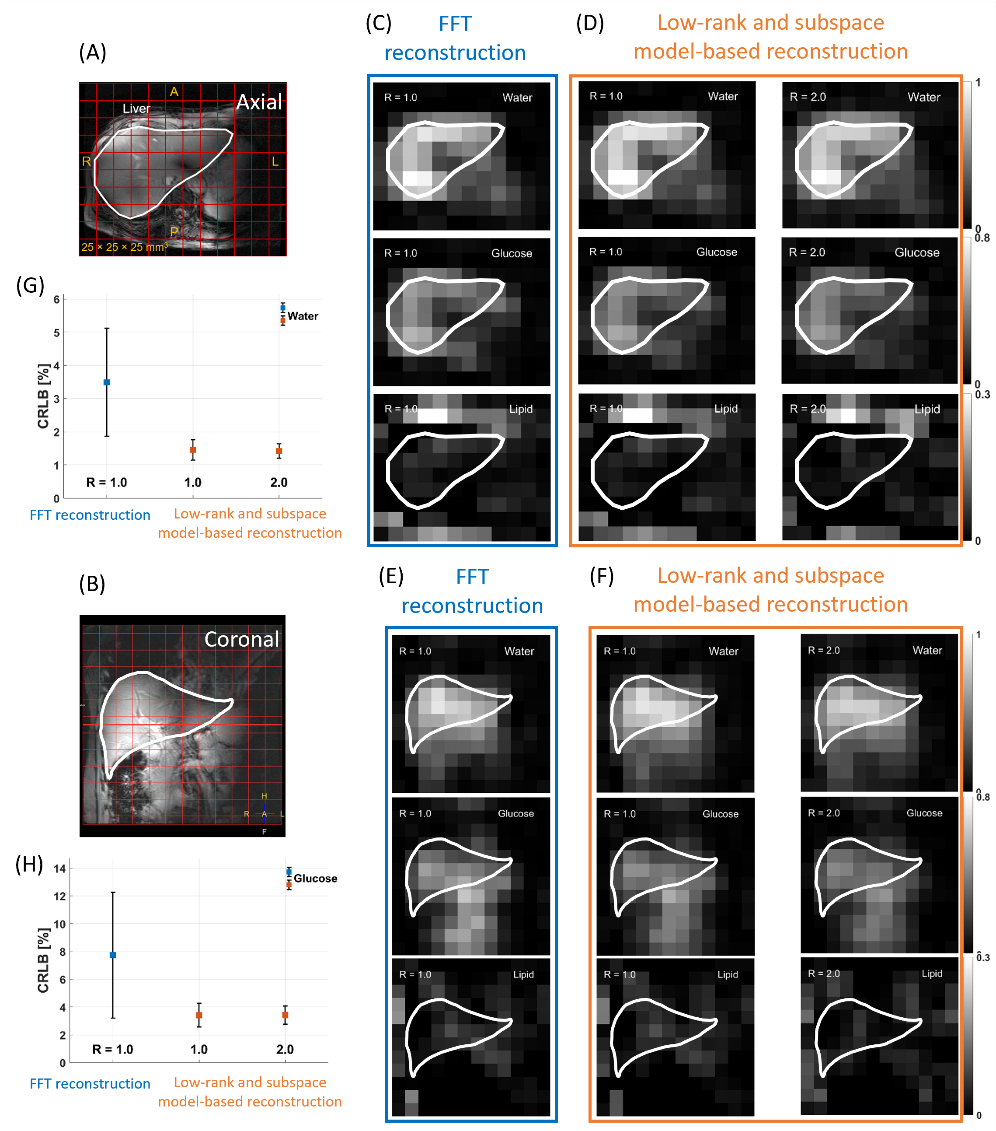


**Figure S4:** Deuterated water, glucose, and lipid metabolite maps derived from DMI data in axial and coronal planes, processed using FFT and low-rank and subspace model-based reconstructions. The ¹H Dixon MRI images show the liver in axial **(A)** and coronal **(B)** views, outlined with a white contour. The metabolite maps present the intensity of glucose and water signals 2.5 hours post oral glucose intake, emphasizing differences between the reconstruction methods. Axial deuterated water, glucose, and lipid maps are displayed for FFT reconstruction **(C)** and low-rank and subspace model-based reconstruction **(D)** at acceleration factors R = 1.0 and 2.0. Similarly, coronal plane metabolite maps are shown for FFT reconstruction **(E)** and low-rank and subspace model-based reconstructions **(F)** at the same acceleration factors. Both methods demonstrate similar distribution patterns for water and glucose maps, even at R = 2.0. While water and glucose maps remain similar at R = 2.0 for both methods, noticeable differences appear in the lipid map when comparing R = 1.0 and R = 2.0 in the low-rank and subspace model-based reconstruction, with lipid signals becoming more variable at higher acceleration. The CRLB [%] values for deuterated water **(G)** and glucose **(H)** were calculated for both methods, with FFT reconstruction showing higher CRLB values across all acceleration factors, indicating reduced fitting accuracy compared to the low-rank approach, particularly for water and glucose signals.
